# CTLA-4-Ig therapy preserves cardiac function following myocardial infarction with reperfusion

**DOI:** 10.1093/cvr/cvaf165

**Published:** 2025-10-03

**Authors:** Jonathan Noonan, Shania A Prijaya, Laura A Bienvenu, Nalin H Dayawansa, Marcel Michla, Viktoria Bongcaron, Prerna Sharma, Yuyang Song, Angela Huang, Anastasia Barbaro-Wahl, Yilu Huang, Hung Nguyen, Anne Nguyen, Andrew J Murphy, Yiyu Zhang, Man K S Lee, Chad J Johnson, Anna M D Watson, Anita C Thomas, James D McFadyen, Daniel G Donner, Xiaowei Wang, Karlheinz Peter

**Affiliations:** Atherothrombosis and Vascular Biology Laboratory, Baker Heart and Diabetes Institute, 75 Commercial Road, Melbourne, VIC 3004, Australia; Baker Department of Cardiometabolic Health, University of Melbourne, Parkville, VIC 3010, Australia; Department of Immunology, School of Translational Medicine, Monash University, 99 Commercial Road, Melbourne, VIC 3004, Australia; Atherothrombosis and Vascular Biology Laboratory, Baker Heart and Diabetes Institute, 75 Commercial Road, Melbourne, VIC 3004, Australia; Baker Department of Cardiometabolic Health, University of Melbourne, Parkville, VIC 3010, Australia; Molecular Imaging and Nanotherapeutics Laboratory, Baker Heart and Diabetes Institute, 75 Commercial Road, Melbourne, VIC 3004, Australia; Molecular Imaging and Nanotherapeutics Laboratory, Baker Heart and Diabetes Institute, 75 Commercial Road, Melbourne, VIC 3004, Australia; Atherothrombosis and Vascular Biology Laboratory, Baker Heart and Diabetes Institute, 75 Commercial Road, Melbourne, VIC 3004, Australia; Department of Medicine, School of Translational Medicine, Monash University, 99 Commercial Road, Melbourne, VIC 3004, Australia; Department of Cardiology, The Alfred Hospital, 55 Commercial Road, Melbourne, VIC 3004, Australia; Atherothrombosis and Vascular Biology Laboratory, Baker Heart and Diabetes Institute, 75 Commercial Road, Melbourne, VIC 3004, Australia; Atherothrombosis and Vascular Biology Laboratory, Baker Heart and Diabetes Institute, 75 Commercial Road, Melbourne, VIC 3004, Australia; Molecular Imaging and Nanotherapeutics Laboratory, Baker Heart and Diabetes Institute, 75 Commercial Road, Melbourne, VIC 3004, Australia; Atherothrombosis and Vascular Biology Laboratory, Baker Heart and Diabetes Institute, 75 Commercial Road, Melbourne, VIC 3004, Australia; Baker Department of Cardiometabolic Health, University of Melbourne, Parkville, VIC 3010, Australia; Molecular Imaging and Nanotherapeutics Laboratory, Baker Heart and Diabetes Institute, 75 Commercial Road, Melbourne, VIC 3004, Australia; Atherothrombosis and Vascular Biology Laboratory, Baker Heart and Diabetes Institute, 75 Commercial Road, Melbourne, VIC 3004, Australia; Atherothrombosis and Vascular Biology Laboratory, Baker Heart and Diabetes Institute, 75 Commercial Road, Melbourne, VIC 3004, Australia; Atherothrombosis and Vascular Biology Laboratory, Baker Heart and Diabetes Institute, 75 Commercial Road, Melbourne, VIC 3004, Australia; Atherothrombosis and Vascular Biology Laboratory, Baker Heart and Diabetes Institute, 75 Commercial Road, Melbourne, VIC 3004, Australia; Molecular Imaging and Nanotherapeutics Laboratory, Baker Heart and Diabetes Institute, 75 Commercial Road, Melbourne, VIC 3004, Australia; Molecular Imaging and Nanotherapeutics Laboratory, Baker Heart and Diabetes Institute, 75 Commercial Road, Melbourne, VIC 3004, Australia; Baker Department of Cardiometabolic Health, University of Melbourne, Parkville, VIC 3010, Australia; Haematopoiesis and Leukocyte Biology Laboratory, Baker Heart and Diabetes Institute, 75 Commercial Road, Melbourne, VIC 3004, Australia; Baker Department of Cardiometabolic Health, University of Melbourne, Parkville, VIC 3010, Australia; Haematopoiesis and Leukocyte Biology Laboratory, Baker Heart and Diabetes Institute, 75 Commercial Road, Melbourne, VIC 3004, Australia; Haematopoiesis and Leukocyte Biology Laboratory, Baker Heart and Diabetes Institute, 75 Commercial Road, Melbourne, VIC 3004, Australia; Bioimaging Platform, La Trobe University, Bundoora, VIC 3083, Australia; Atherothrombosis and Vascular Biology Laboratory, Baker Heart and Diabetes Institute, 75 Commercial Road, Melbourne, VIC 3004, Australia; Department of Diabetes, School of Translational Medicine, Monash University, 99 Commercial Road, Melbourne, VIC 3004, Australia; Translational Cardiology Centre, Baker Heart and Diabetes Institute, 75 Commercial Road, Melbourne, VIC 3004, Australia; Heart Centre, The Alfred Hospital, 55 Commercial Road, Melbourne, VIC 3004, Australia; Atherothrombosis and Vascular Biology Laboratory, Baker Heart and Diabetes Institute, 75 Commercial Road, Melbourne, VIC 3004, Australia; Baker Department of Cardiometabolic Health, University of Melbourne, Parkville, VIC 3010, Australia; Department of Clinical Haematology, School of Translational Medicine, Monash University, 99 Commercial Road, Melbourne, VIC 3004, Australia; Australian Centre for Blood Diseases, School of Translational Medicine, Monash University, 99 Commercial Road, Melbourne, VIC 3004, Australia; Baker Department of Cardiovascular Research, Translational and Implementation, La Trobe University, Bundoora, VIC 3083, Australia; Baker Department of Cardiometabolic Health, University of Melbourne, Parkville, VIC 3010, Australia; Department of Medicine, School of Translational Medicine, Monash University, 99 Commercial Road, Melbourne, VIC 3004, Australia; Translational Cardiology Centre, Baker Heart and Diabetes Institute, 75 Commercial Road, Melbourne, VIC 3004, Australia; Heart Centre, The Alfred Hospital, 55 Commercial Road, Melbourne, VIC 3004, Australia; Baker Department of Cardiometabolic Health, University of Melbourne, Parkville, VIC 3010, Australia; Molecular Imaging and Nanotherapeutics Laboratory, Baker Heart and Diabetes Institute, 75 Commercial Road, Melbourne, VIC 3004, Australia; Department of Medicine, School of Translational Medicine, Monash University, 99 Commercial Road, Melbourne, VIC 3004, Australia; Baker Department of Cardiovascular Research, Translational and Implementation, La Trobe University, Bundoora, VIC 3083, Australia; Atherothrombosis and Vascular Biology Laboratory, Baker Heart and Diabetes Institute, 75 Commercial Road, Melbourne, VIC 3004, Australia; Baker Department of Cardiometabolic Health, University of Melbourne, Parkville, VIC 3010, Australia; Department of Immunology, School of Translational Medicine, Monash University, 99 Commercial Road, Melbourne, VIC 3004, Australia; Department of Medicine, School of Translational Medicine, Monash University, 99 Commercial Road, Melbourne, VIC 3004, Australia; Department of Cardiology, The Alfred Hospital, 55 Commercial Road, Melbourne, VIC 3004, Australia; Baker Department of Cardiovascular Research, Translational and Implementation, La Trobe University, Bundoora, VIC 3083, Australia

**Keywords:** Myocardial infarction, Inflammation, Ultrasound

## Abstract

**Aims:**

T cells drive adverse cardiac inflammation and ischemia-reperfusion injury following myocardial infarction (MI). Here, we aimed to test the extent to which T cell inhibition protected cardiac function following MI in mice.

**Methods and results:**

Cardiac ischemia-reperfusion injury (CIRI), mimicking MI with successful reperfusion therapy, was induced in C57BL/6J mice via temporary surgical ligation of the left anterior descending artery. T cell inhibition was achieved using abatacept, an FDA-approved CTLA-4-Ig fusion protein. Multiple treatment strategies were assessed, ranging from prolonged treatment across 4 weeks to short-term treatment, also with delayed time-to-intervention. Cardiac function was assessed using echocardiography, including strain analysis. Impacts on the cardiac and systemic immune response were assessed using flow cytometry. CIRI-induced robust CD4^+^ biased T cell activation in the heart within 7 days. Treatment with abatacept significantly preserved key echocardiographic metrics of cardiac function. This treatment coincided with near-complete inhibition of the cardiac T cell response, as well as reductions in innate inflammatory cells. Collectively, this demonstrated a central mechanistic role for T cell activation post-MI with reperfusion. Evaluation of short-term intervention strategies further demonstrated sustained preservation of cardiac function even where treatment was delayed by 24 h. Mechanistically, our data indicate that over 50% of lost cardiac function post-MI with reperfusion is T cell dependent.

**Conclusion:**

T cell co-stimulation leading to activation is a central driver of the cardiac immune response following MI with reperfusion. The inhibition of this axis significantly protected against CIRI and preserved cardiac function. Ultimately, we highlight T cell immunomodulation and abatacept as highly promising approaches for clinical translation.

## Graphical Abstract

**Graphical Abstract:**
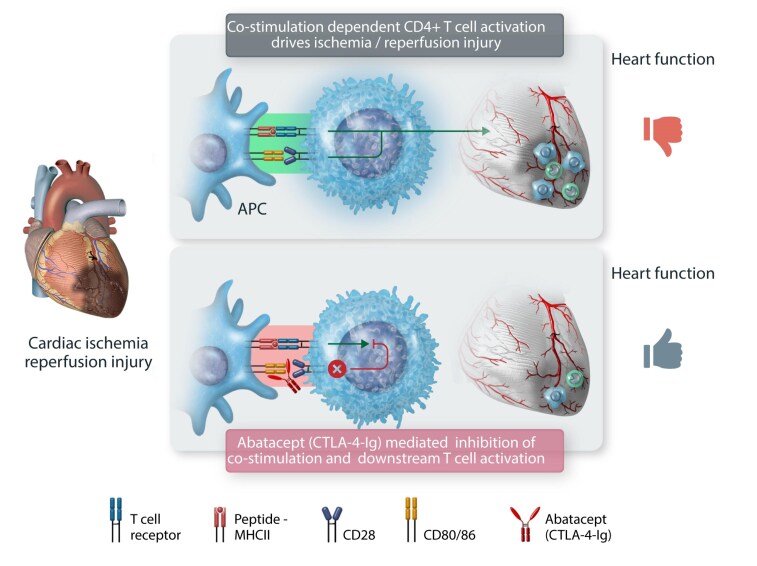

**Time of primary review: 71 days**

## 1. Introduction

Myocardial infarction (MI) most often manifests from the rupture of unstable atherosclerotic plaques, leading to the thrombotic occlusion of coronary arteries.^1,2^ Major progress in percutaneous coronary intervention (PCI) and systems of care has allowed for rapid restoration of coronary flow in patients with acute MI, leading to significant improvements in survival. However, myocardial injury and necrosis continue after reperfusion in the form of cardiac ischemia-reperfusion injury (CIRI), where inflammation plays a critical role, driving loss of heart function and progression towards heart failure. To date, no interventions have been able to address this paradoxical overshooting inflammation in reperfused myocardium, with the 1-year mortality of patients following ST-elevation MI (STEMI) remaining as high as 14%.^3,4^ Consequently, cardiac inflammation and CIRI represent key and yet unaddressed targets for improving MI treatment.^5–7^

T cells are central mediators of inflammation, capable of producing an array of pro- and anti-inflammatory effector functions including direct cell killing and cytokine production. Following MI in mice, effector/memory T cells (T_E/M_) are recruited to the heart, where they cause damage and promote adverse cardiac remodelling.^8,9^ In contrast, although less abundant, CD4^+^ FoxP3^+^ regulatory T cells (T_regs_) function to limit cardiac inflammation. Global T cell deficiency has been shown to reduce cardiac scar formation in mice post-MI, while T_reg_ ablation exacerbates cardiac damage,^9^ highlighting a delicate immunological balance between pro-inflammatory and immunoregulatory T cell–mediated functions. Moreover, the concept that ischemic heart disease contains a strong autoimmune component has gained increasing traction over recent years.^10,11^

Despite emerging pre-clinical evidence that T cells play a central role in cardiac damage in multiple contexts, limited research has explored their therapeutic targeting post-MI. Moreover, it is unclear how pathways critical to T cell activation and function influence CIRI. A key focus of this study is CTLA-4 (CD152; cytotoxic T-lymphocyte associated protein 4), an immune checkpoint molecule expressed by T cells following their activation to limit their function and protect from aberrant activation/inflammation.^12^ Mechanistically, CTLA-4’s high affinity for CD80 and CD86, expressed by antigen-presenting cells, prevents their interaction with CD28 on the T cell surface, an interaction that would otherwise promote activation. Immune checkpoint inhibition (ICI) therapies, including those blocking CTLA-4, promote T cell responses and have proven to be an excellent therapy for several cancers. However, both clinical and pre-clinical studies show that cardiovascular inflammation and increased risk of MI are major and life-threatening side effects of ICI mediated T cell activation.^13–18^ In contrast, by functionally mimicking endogenous CTLA-4, the CTLA-4-Ig fusion protein abatacept has revolutionized the treatment of rheumatoid arthritis by limiting T cell–driven inflammation.^19^ Evidence now also supports that abatacept treatment reduces cardiovascular risk in rheumatic patients, blunts age-related and surgically induced heart failure in mice, reduces oxidative stress and apoptosis in a rat model of chemically induced myocardial necrosis with a perspective demonstrating improved haemodynamics in CIRI, and has been highlighted as a solution to ICI myocarditis.^20–26^ Combined, these data thus suggest that T cell responses are broadly detrimental following cardiac injury, and that abatacept (and CTLA-4 itself) is broadly cardioprotective.

Collectively, current data suggest that T cell responses play a central role in the regulation of cardiovascular inflammation. As such, modulating checkpoint proteins such as CTLA-4 represents an attractive target to limit maladaptive T cell responses therapeutically post-MI. Here, we provide proof of concept that inhibiting T cell activation using abatacept reduces cardiac inflammation and preserves cardiac function following experimental CIRI. In doing so, we highlight the mechanistic importance of co-stimulation dependent T cell responses to CIRI and demonstrate the significant potential for using abatacept as a novel and promising MI treatment.

## 2. Methods

Expanded methodologies can be found within the *Supplementary Materials*.

### 2.1 Mice

Experiments used 6–10-week-old male and female C57BL/6J mice (Jackson Laboratories, USA). All experiments involving animals were ethically approved and conformed to the guidelines under Directive 2010/63/EU of the European Parliament on the protection of animals used for scientific purposes.

### 2.2 CIRI model

MI was induced as previously described.^27^ Briefly, animals were anesthetized, endotracheally intubated and provided with analgesia. The thoracotomy region was exposed and a polypropylene (7–0) suture tied through two exteriorized releasing loops was used to ligate and occlude the left anterior descending (LAD) artery. The chest cavity and skin were then closed. Reperfusion was induced after 60 min by pulling on exteriorized loops. Mice were euthanised using ketamine (300 mg/kg) and xylazine (30 mg/kg).

### 2.3 Abatacept treatment

Mice were randomized and then abatacept (Bristol Myer Squibb; 10 mg/kg) or vehicle control (0.9% sodium chloride) was injected i.p. according to defined treatment strategies.

### 2.4 Echocardiography

Mice were placed under light sedation with 1–2% isoflurane. Ultrasound was performed using a Vevo2100 high-resolution small-animal scanner. The parasternal short and/or long-axis views of the heart were imaged. Imaging and analysis were performed in a double-blinded manner.

### 2.5 Flow cytometry

Cells isolated from all tissues were stained with fixable viability dye and fluorochrome-conjugated antibodies. Data were acquired using a BD LSR Fortessa X-20 (BD Bioscience, USA). Data analysis was performed using FlowJo (Flowjo LLC, USA). A complete list of antibodies/dyes used is provided in Supplementary material online, *Table S1*. An overview of analytical gating is shown in Supplementary material online, *Figure S1*.

### 2.6 Immunohistochemistry

Paraffin sections were stained with anti-CD3e antibody and CD68 and then visualized with 3,3 Diaminobenzidine tetrahydrochloride.

### 2.7 Analysis of cardiac fibrosis by histology

Paraffin sections of the left ventricle (LV) were stained with Picrosirius Red (PSR) to label fibrotic tissue. The percentage of fibrosis was calculated as the percentage of total PSR area against total LV tissue area.

### 2.8 Gene expression analysis of cardiac leukocytes

CD45^+^ cells were purified from mouse hearts using magnetic-bead positive selection. RNA was then extracted using TRIzol. Real-time quantification of gene expression was performed, and the relative expression of each gene normalized to GAPDH.

### 2.9 Statistics

All statistical analyses were performed using GraphPad Prism (Version 10.1.1). Outlier testing was conducted followed by normality testing. Data were then analysed with statistical tests appropriate for the number of groups, normality, and variance.

## 3. Results

### 3.1 Myocardial infarction with reperfusion induces robust cardiac T cell activation

**Figure 1:**
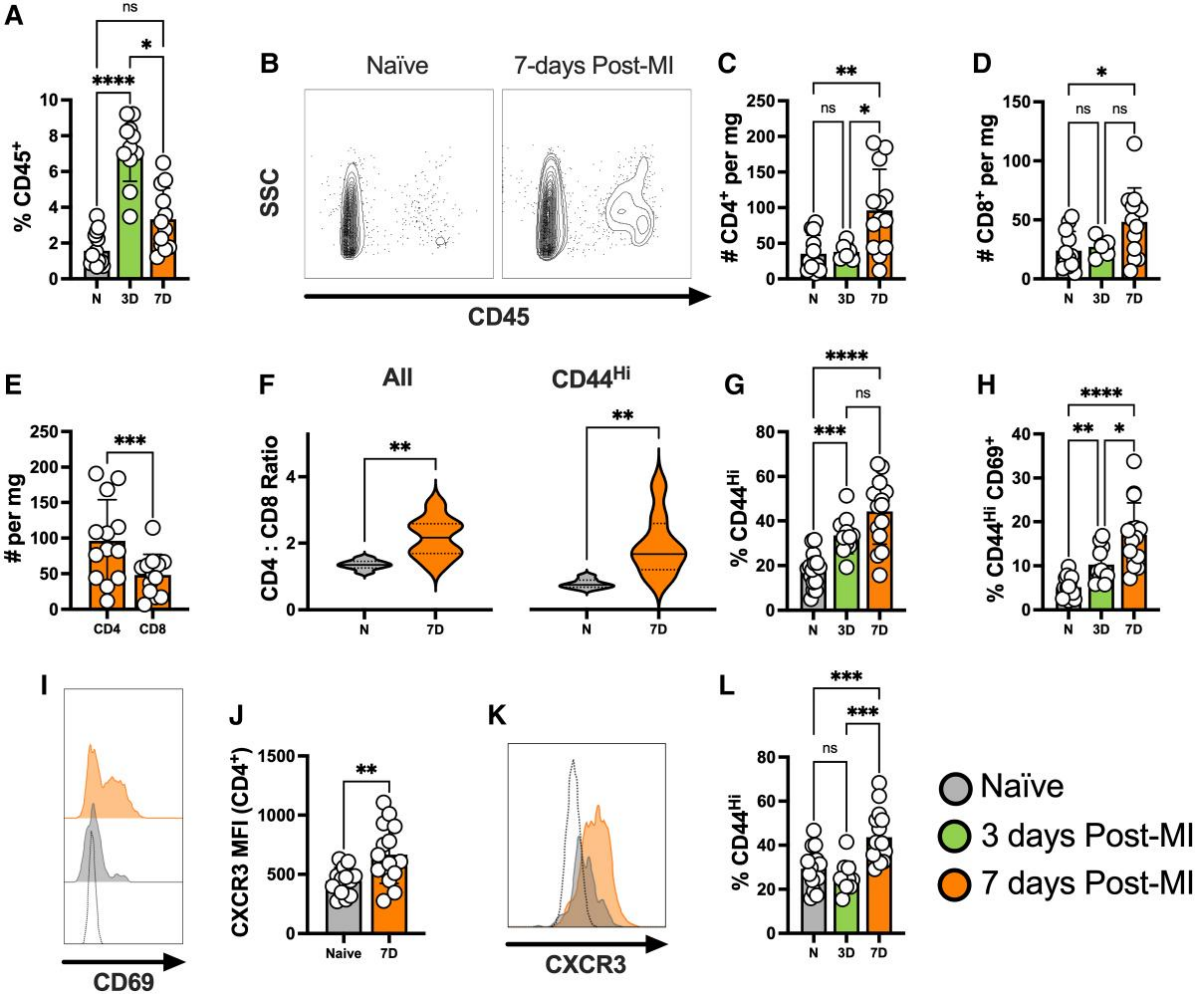
Myocardial infarction with reperfusion rapidly induces a local cardiac T cell response. C57BL/6J mice were subjected to temporary LAD ligation and the local cardiac immune response was characterized 3- and 7- days following injury using flow cytometry. Naïve mice that did not receive LAD ligation were included as controls. Proportions of CD45^+^ leukocytes (*A*) alongside representative plots (*B*). Absolute numbers of CD4^+^ (*C*) and CD8^+^ T cells (*D*) per mg of heart tissue. Comparison of absolute CD4^+^ and CD8^+^ T cell numbers 7 days post-injury (*E*) alongside CD4:CD8 ratios for all T cells and CD44^Hi^ T cells (*F*). Proportions of CD4^+^ T cells exhibiting a CD44^Hi^ effector/memory phenotype (*G*) and those co-expressing CD44 and CD69 (*H*) with a representative CD69 expression histogram (*I*). CXCR3 mean fluorescence intensity for CD4^+^ T cells (*J*) and a representative histogram (*K*). Proportions of CD8^+^ T cells exhibiting a CD44^Hi^ effector/memory phenotype (*L*). In both (*I* and *K*), the fluorescent minus one control is shown as a transparent histogram. *n* represents the number of biological replicates, which are graphed as individual points where possible. *n =* 11–15 (*A*, *G*, *H*, *J*, *L*); *n =* 6–8 (*C*, *D*, *E*, *F*). Statistics used: Kruskal-Wallis (*A*), Brown-Forsythe and Welch ANOVA (*C*, *D*, *G*, *H*), paired *t*-test (*E*), Welch’s *t*-test (*F*), unpaired *t*-test (*J*) and ordinary one-way ANOVA (*L*) statistical tests were performed. *P* < 0.05*; *P* < 0.01**; *P* < 0.001***, *P* < 0.0001****, ns = not significant. N = Naïve, 3D = 3 days post-MI, 7D = 7 days post-MI.

To characterize the T cell response to CIRI, mice underwent temporary LAD ligation and their cardiac immune cell profiles were assessed using flow cytometry. Consistent with published data,^28^ the representation of cardiac leukocytes was significantly increased post-CIRI, peaking at ∼3 days (*Figure 1A* and *B*). In keeping with typical adaptive immune dynamics, CD4^+^ and CD8^+^ T cell numbers increased within the infarcted heart 7-days following injury (*Figure 1C* and *D*). Comparing these, we found markedly higher numbers of CD4^+^ vs. CD8^+^ T cells in the heart, resulting in an elevated CD4:CD8 ratio for all T cells and those with an antigen-experienced effector/memory phenotype (CD44^Hi^, T_E/M_; *Figure 1E* and *F*). Due to the skewed enrichment for CD4^+^ T cells we focused our investigations in their direction.

We next identified that increased proportions of CD4^+^ T_E/M_ within the heart were detectible as early as day 3 post-CIRI, being further enriched by day 7 (*Figure 1G*). T cell activation was also reflected in co-expression of CD69, a marker of recent activation and/or tissue residency (*Figure 1H* and *I*). Cardiac CD4^+^ T cells are typically skewed toward a pro-inflammatory T_H_1 phenotype following MI, which was confirmed by increased expression of CXCR3, a T_H_1 associated chemokine receptor^29^ (*Figure 1J* and *K*). This also highlighted biased recruitment of effector T cells, as regulatory cells strongly express ST2 and not CXCR3 post-MI (ref^30^ and data not shown). CD8^+^ T cells with a T_E/M_ phenotype were also significantly increased by day 7 post-CIRI (*Figure 1L*). Collectively, these findings show that T cells are strongly activated in the heart following CIRI and, given T cells’ functional capacity, indicates their likely importance as a therapeutic target.

### 3.2 Abatacept treatment preserves heart function post-MI

**Figure 2:**
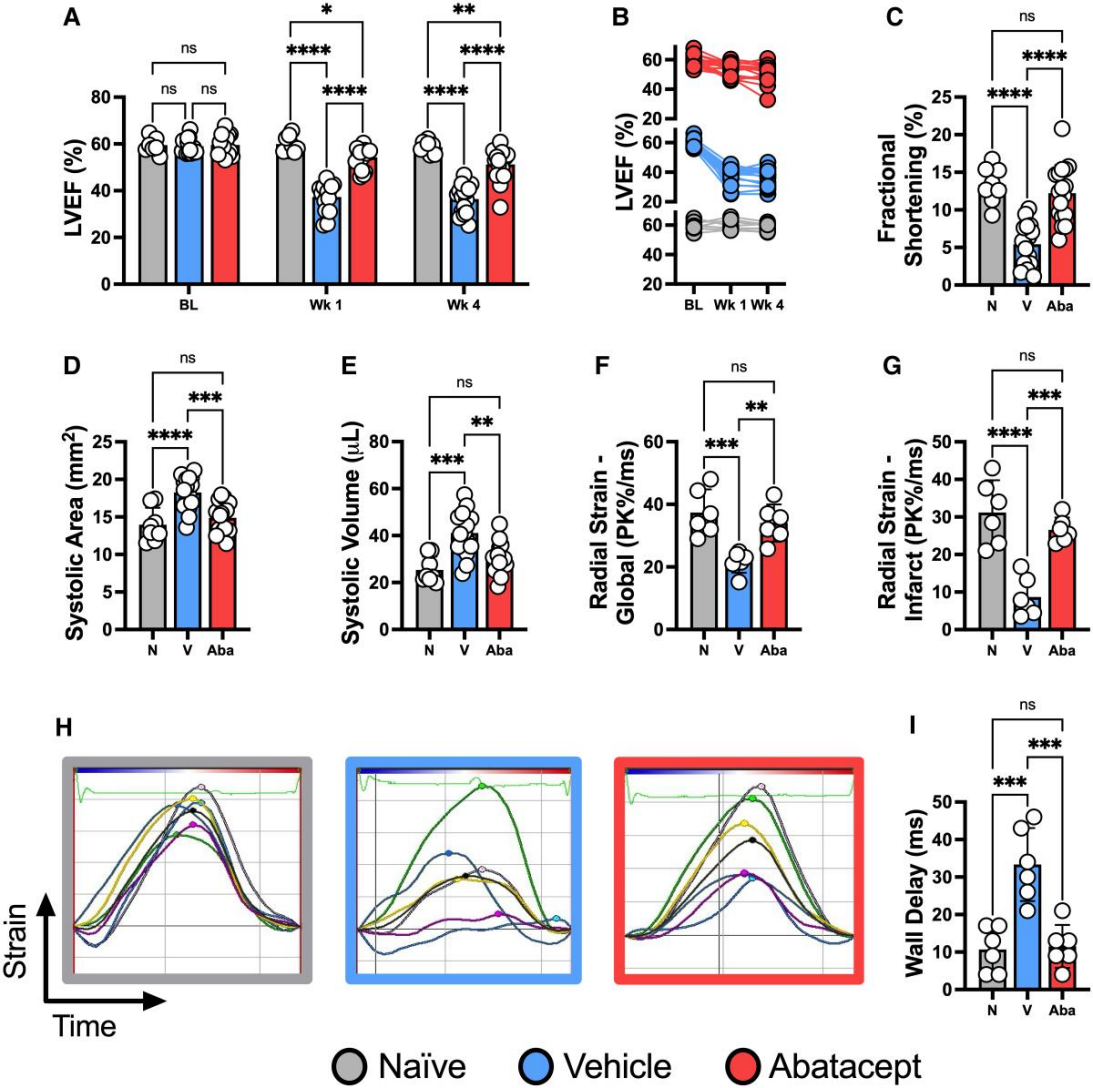
Abatacept preserves cardiac function following MI with reperfusion. C57BL/6J mice were subjected to temporary LAD ligation and then treated from the time of reperfusion with abatacept or vehicle 3× per week for 4 weeks. Naïve mice that did not undergo LAD ligation were included as controls. Left ventricular ejection fraction (LVEF) is shown at baseline, 1 and 4 weeks post-MI as a summary bar chart (*A*) and line graph to demonstrate longitudinal function per mouse (*B*). All subsequent graphs are from the 4-week post-MI time point, including fractional shortening (*C*), systolic area (*D*), and systolic volume (*E*). Radial strain analysis is presented globally (*F*) across the whole left ventricle and for the anteroapical infarct site (*G*). Representative radial strain curves; coloured lines represent each assessed myocardial region and black lines represent the mean (*H*). Maximum opposite wall delay time (*I*). *n* represents the number of biological replicates, which are graphed as individual points where possible. *n =* 8–17 (*A–E*); *n =* 6 (*F*, *H*, *I*). Statistics used: Mixed-effects (*A*), Kruskal-Wallis (*E*) and ordinary one-way ANOVA tests (*C*, *D*, *F*, *G*, *I*) were performed. *P* < 0.05*; *P* < 0.01**; *P* < 0.001***; *P* < 0.0001****, ns = not significant. N = Naïve, V = Vehicle, Aba = Abatacept.

Abatacept is an FDA-approved CTLA-4-Ig fusion protein that robustly inhibits co-stimulatory signals critical for T cell activation.^12^ Abatacept is used clinically as an effective and extremely well characterized T cell inhibitor in the setting of autoimmunity, and remains a prototypical pre-clinical tool for the precise inhibition of T cell responses.^19,31,32^ As such, we used abatacept to simultaneously gain mechanistic insights into the importance of co-stimulation dependent T cell responses in CIRI and assess the therapeutic value of T cell inhibition. To identify if CTLA-4–mediated T cell modulation is protective post-MI, mice were randomized and treated with abatacept from the time of reperfusion at a clinically approved dosage (10 mg/kg^19^) or vehicle control. The initial frequency of dosing, 3× per week, was adapted from pre-clinical studies of rheumatoid arthritis.^31,32^ Naïve mice that did not undergo surgery were included to allow for comparisons to healthy heart function. We identified that abatacept-treated mice had significant and sustained protection of cardiac function within 1-week of the ischemic event (*Figure 2A* and *B*). At 4 weeks post-MI, vehicle control mice demonstrated a nearly 40% relative reduction in LVEF from baseline. In contrast, abatacept-treated mice showed a significantly attenuated functional deficit, with fractional shortening, systolic area, and systolic volume all preserved in abatacept-treated mice (*Figure 2C–E*).

We next conducted strain analysis, a powerful approach in clinical cardiac ultrasound with high sensitivity for cardiac dysfunction.^33^ In this analysis, the left ventricle is computationally segmented into distinct anatomical regions, the base, middle and apex of the anterior and posterior wall. Each segment contains multiple data points that track the dynamics of muscle contraction (see Supplementary material online, *Figure S2*; tracking of muscle contraction can be seen in Supplementary material online, *Videos S1*–S5). In vehicle-treated mice, we observed significant abnormalities in contractile movement and left ventricle dysfunction. This was prevented by abatacept treatment both globally and specifically at the major site of damage, the anterior apex (AA) (*Figure 2F* and *G*). Representative traces of these abnormalities are shown in *Figure 2H*, where non-gaussian (asynchronous) patterns of contraction are observed only in vehicle-treated animals’ post-CIRI. Abatacept further prevented increases to maximum opposite wall delay time, reflecting effective cooperation between the anterior and posterior walls (*Figure 2I*). In addition to demonstrating robust protection of cardiac function by abatacept, these data further implicate CTLA-4 as a powerful cardioprotective protein in the context of reperfused MI.

### 3.3 Abatacept treatment inhibits MI-induced immune responses

**Figure 3:**
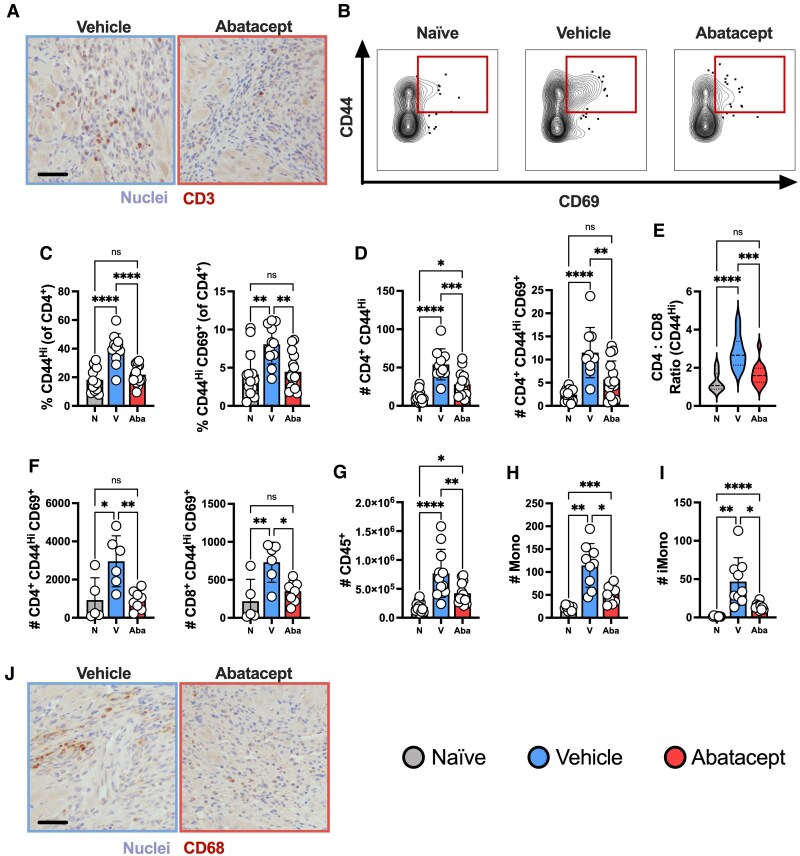
Abatacept suppresses the immune response post-MI. C57BL/6J mice were subjected to temporary LAD ligation and then treated from the time of reperfusion with abatacept or vehicle 3× per week. Naïve mice that did not receive LAD ligation were included as controls. All analyses are from 7 days post-MI. Immunohistochemical staining of CD3 in the heart (*A*). Representative plots of CD44 and CD69 co-expression (*B*). Proportions of cardiac CD4^+^ T cells exhibiting a CD44^Hi^ effector/memory phenotype or co-expressing CD44 and CD69 (*C*). Quantification of absolute numbers per mg from C (*D*). Ratio of cardiac CD4 to CD8 cells with a CD44^Hi^ effector/memory phenotype (*E*). Absolute numbers of CD4^+^ and CD8^+^ T cells co-expressing CD44 and CD69 in the mediastinal lymph node (*F*). Absolute numbers of total cardiac leukocytes (CD45^+^) per heart alongside (*G*), total monocytes (*H*), and ‘inflammatory’ Ly6C^Hi^ monocytes [iMono] (*I*) per mg of heart tissue. Immunohistochemical staining of CD68 in the heart (*J*). Scale bars = 50μm and apply to all representative images. *n* represents the number of biological replicates, which are graphed as individual points where possible. *n =* 10–15 (*B–E*, *G*); *n =* 5–8 (*F*); *n* = 9–11 (*G–I*). Statistics used: ordinary one-way (*B–G*) and Brown-Forsythe and Welch (*H–I*) ANOVA tests were performed. *P* < 0.05*; *P* < 0.01**; *P* < 0.001***; *P* < 0.0001****, ns = not significant. N = Naïve, V = Vehicle, Aba = Abatacept.

We then tested whether abatacept treatment inhibited local and/or systemic T cell responses. Immunohistochemical staining of CD3 identified that T cells were found within highly nucleated sites of cardiac damage post-CIRI, but rarely within healthy myocardium, and appeared reduced within the hearts of abatacept-treated mice (*Figure 3A*). To quantitatively assess these changes to the T cell response at high-resolution we used flow cytometry, identifying that abatacept dramatically reduced the representation of activated CD4^+^ T_E/M_ in the heart 7-days post-MI, including those co-expressing CD69 (*Figure 3B* and *C*). These proportional reductions were also reflected in absolute T cell numbers (*Figure 3D*). We additionally identified a significant reduction in the CD4:CD8 T_E/M_ ratio to near-naïve levels, further demonstrating a blunting of this CD4^+^ biased response (*Figure 3E*). Going beyond the heart, T cell inhibition extended to both CD4^+^ and CD8^+^ T cells in the draining mLN (*Figure 3F*).

T cells play a critical role in orchestrating the immune response, including directing the recruitment and function of innate immune cells. Consequently, we hypothesized that CTLA-4–mediated T cell inhibition would lead to a downstream dampening of the total leukocyte response post-MI, including reductions in innate immunity. Supporting this, we identified total leukocyte numbers were reduced in the heart following abatacept treatment (*Figure 3G*), as well as total and inflammatory (Ly6C^Hi^) monocytes (*Figure 3H* and *I*). CD68^+^ staining (predominantly inflammatory monocytes), mostly found within highly nucleated areas of damage, was also markedly reduced in abatacept-treated animals (*Figure 3J*). Combined, these data demonstrate abatacept‛s ability to shut down T cell activation post-CIRI, as intended, leading to significant reductions in the immune response both locally and in the draining mLN.

### 3.4 Short-term treatment with abatacept is sufficient for cardio-protection post-MI

**Figure 4:**
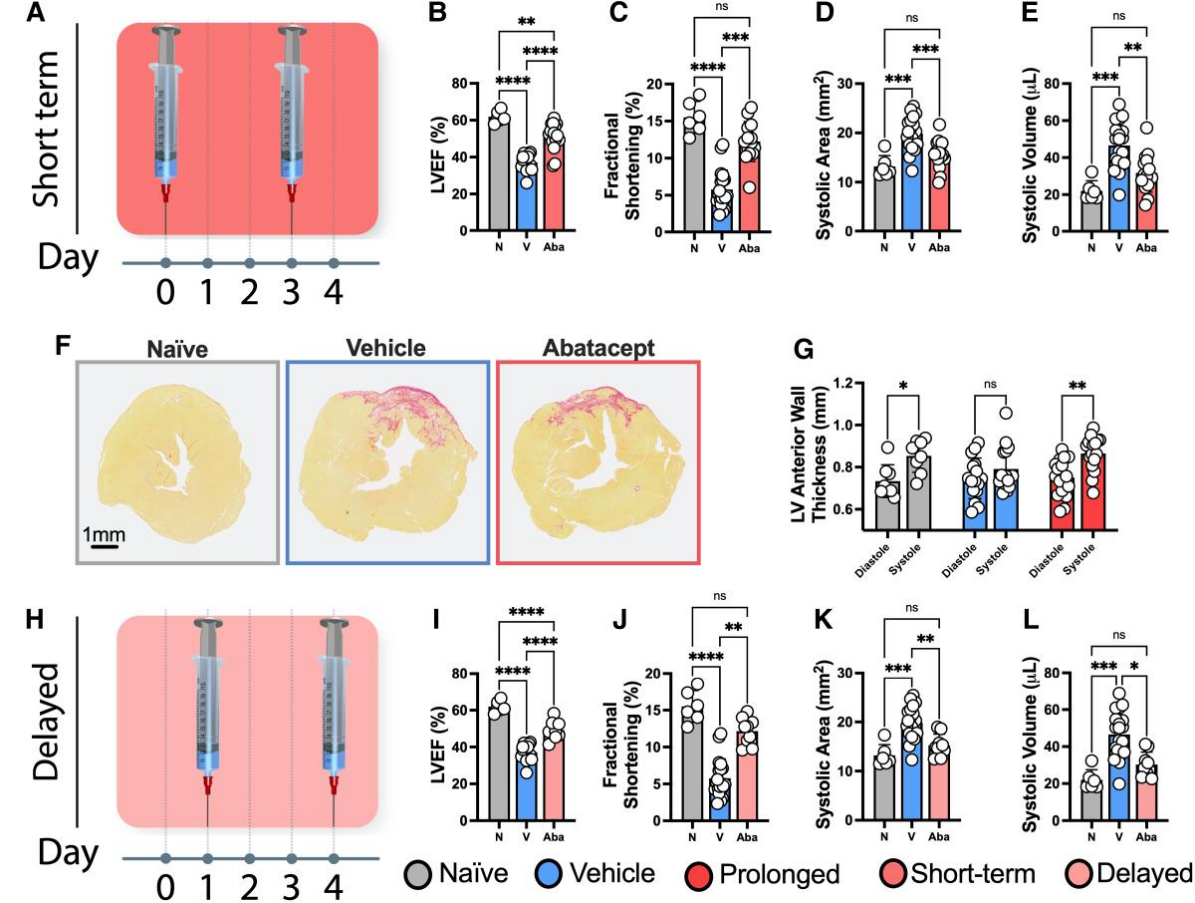
Short-term and delayed abatacept treatment is sufficient for cardio-protection post-MI. C57BL/6J mice were subjected to temporary LAD ligation and then treated with abatacept or vehicle using a short-term (*A–F*) treatment regime. Naïve mice not subjected to LAD ligation were included as controls. Mice then underwent echocardiography 4 weeks post-MI. A schematic of the short-term treatment strategy (*A*) with associated LVEF (*B*), fractional shortening (*C*), systolic area (*D*), and systolic volume (*E*). PSR staining of fibrosis in the mid-ventricle of excised hearts (*F*). Scale bar = 1 mm and applies to all representative images. Paired analysis of LV anterior wall thickness in mice from the prolonged abatacept treatment group from *Figure 2* (*G*). In concert, we assessed the impact of delaying abatacept treatment to day 1 (24 h post-MI) and day 4 post-MI, analysing the same functional echocardiographic metrics as above (*H*–*L*). *n* represents the number of biological replicates, which are graphed as individual points. *n =* 6–19. Statistics used: ordinary one-way ANOVA (*B*, *D*, *I*, *K*), Kruskal-Wallis (*C*, *E*, *J*, *L*) and mixed effects analyses (*G*) were performed. *P* < 0.05*; *P* < 0.01**; *P* < 0.001***, *P* < 0.0001****, ns, not significant; N, Naïve; V, Vehicle; Aba, Abatacept.

The adaptive immune response typically peaks days to weeks after the maximal innate response. This pattern is at least partially mediated by the requirement for adaptive immune cells to interact with innate immune cells for their activation. Given that the peak of the cardiac innate response occurs ∼3 days post-MI,^28^ we hypothesized that short-term treatment with abatacept within 7 days of MI would be sufficient to confer protection. Our rationale was that, following this period, there would be reduced possibility for the innate system to prime an adaptive response. Treating mice at the time of reperfusion and on day 3 (*Figure 4A*), we again identified near-complete preservation of cardiac function, including LVEF, Fractional Shortening, Systolic Area and Systolic Volume (*Figure 4B–E*).

Cardiac remodelling, including the formation of a fibrotic scar to replace dead cardiomyocytes, is critical to prevent rupture of the heart muscle post-MI. However, the subsequent expansion of fibrosis is also then a key driver of further reduced cardiac function. Investigating this, we found that hearts appeared larger 28 days post-CIRI, consistent with injury induced remodelling. However, this increase reached significance only in the vehicle group (see Supplementary material online, *Figure S3A*). Moreover, we detected the same pattern of response in heart to body weight ratios by D7 post-MI, overall suggesting that remodelling is partially blunted in the abatacept-treated group (see Supplementary material online, *Figure S3B*). However, we found no significant differences in the representation of fibrosis, with hearts from both vehicle and abatacept groups presenting with clear cardiac scarring (*Figure 4F* and Supplementary material online, *Figure S3C*). While this could appear surprising given the improved function, several other studies have also demonstrated changes to cardiac function without major impacts on fibrotic burden, with inflammation heavily implicated in this phenomenon.^20,34–36^ Thus, while it did not reduce the core infarct area, abatacept appears to attenuate remodelling and dysfunction within the remaining viable myocardium.

To then further assess the heart, we returned to our prolonged treatment data, where we had additionally performed short axis echocardiographic imaging. In doing so, we found that vehicle-treated mice had reduced left ventricular thickening in the anterior wall during systole (i.e. during contraction) in comparison to abatacept-treated mice 28-days post-CIRI (*Figure 4G* and Supplementary material online, *Figure S3D–F*). Moreover, we identified this reduced function in vehicle-treated mice was also detected in the posterior wall of the left ventricle (see Supplementary material online, *Figure S3G* and *H*). As such, these data highlight that T cell inhibition improves contractility in both the infarcted LAD-supplied territory as well as territories outside of the infarct zone.

Going further, we hypothesized that delaying abatacept treatment post-CIRI would not compromise its therapeutic benefit given the slower dynamics of the adaptive response. To test this, we delayed the administrations of abatacept by 24 h (*Figure 4H*), again observing significant improvements in cardiac function (*Figure 4I–L*).

Collectively, these results demonstrate the importance of the CD28:CD80/86 T cell co-stimulatory pathway to T cell activation and loss of heart function following MI with reperfusion. Critically important for translation, these data suggest that short-term treatment with abatacept is sufficient to mediate lasting protection. Given that the time from symptom onset to hospitalization on average is often within 4 h,^37^ this provides a promising window in which abatacept could be given to patients presenting with MI during their routine cardiac care and prior to discharge (typically ∼3 days post-MI).

**Figure 5:**
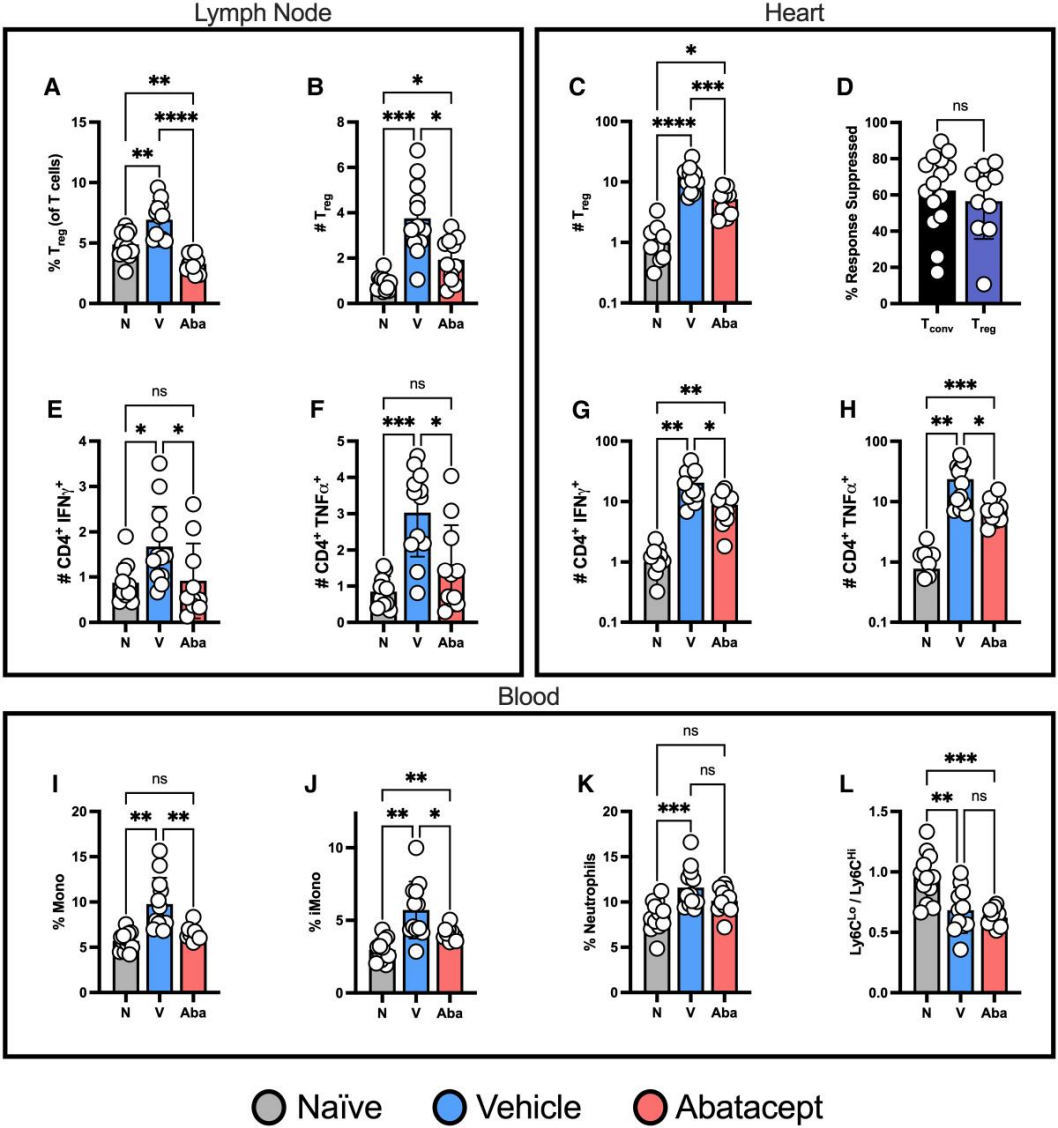
Abatacept prevents cardiac T cell mediated pro-inflammatory cytokine production and systemic monocyte activation post-CIRI. C57BL/6J mice were subjected to temporary LAD ligation and then treated with abatacept or vehicle using a short-term treatment regime. Naïve mice not subjected to LAD ligation were included as controls. All analyses are from 7 days post-CIRI. Proportions (*A*) and numbers (*B*) of CD4^+^ FoxP3^+^ regulatory T cells (T_reg_) in the mLN. Numbers of T_reg_ in the Heart (*C*) and the proportion of their response inhibited vs. non-regulatory conventional T cells (T_conv_; *D*). Numbers of activated IFNγ (*E*) and TNFα (*F*) producing CD4^+^ CD44^+^ CD69^+^ T cells in the mLN. Numbers of activated IFNγ (*G*) and TNFα (*H*) producing CD4^+^ CD44^+^ CD69^+^ T cells in the Heart. Proportion of total monocytes (*I*), Ly6C^Hi^ monocytes (*J*), neutrophils (*K*) and the ratio of ‘reparative’ Ly6C^Lo^ to ‘inflammatory’ Ly6^Hi^ monocytes (*L*) in peripheral blood. *n* represents the number of biological replicates, which are graphed as individual points where possible. *n =* 9–15. Statistics used: ordinary one-way (*L*) and Brown-Forsythe and Welch (*A–C*, *E–L*) ANOVA and unpaired *t*-tests (*D*) were performed. *P* < 0.05*; *P* < 0.01**; *P* < 0.001***; *P* < 0.0001****, ns = not significant. N = Naïve, V = Vehicle, Aba = Abatacept.

In contrast to the detrimental contributions of pro-inflammatory T cell subsets, T_regs_ can play a highly protective role following MI.^38^ However, abatacept is known to suppress T_reg_ responses as they also rely on CD28:CD80/86 signalling for survival and proliferation.^39^ We identified that this was also the case following CIRI, with short-term abatacept treatment preventing the expansion of T_regs_ in the mLN (*Figure 5A* and *B*) and heart (*Figure 5C*) to the same extent as conventional (FoxP3^−^) T cells (*Figure 5D*).

To gain further insight into how abatacept changes the inflammatory environment to mediate protection, we next assessed cytokine production by T cells following CIRI. In the mLN, we identified that abatacept prevented the generation of activated IFNγ, TNFα and IL17A producing T cells (*Figure 5E* and *F* & Supplementary material online, *Figure S4A*). Moreover, while technical challenges prohibited the assessment of IL17A, reductions in IFNγ and TNFα producing T cells were also a strong feature in the heart (*Figure 5G* and *H*). While the contribution of IL17 cytokines to cardiac pathology remains a subject of debate, TNFα and IFNγ are both known drivers of cardiac injury implicated in loss of heart function.^40–42^ As such, preventing the production of these pro-inflammatory cytokines by T cells provides a clear mechanistic avenue by which abatacept can preserve heart function.

Pro-inflammatory cytokine production also strongly influences the mobilization, recruitment and differentiation of innate immune cells,^43,44^ in part by inducing the expression of chemokines. Following CIRI, we identified that cardiac leukocytes expressed significantly less *CXCL2*, *CCL3* and *CCL4* (but not *CCL2* or *CCL5*) following abatacept treatment (see Supplementary material online, *Figure S4B*). This then explains, at least in part, the reduced innate immune cell recruitment to the heart presented in *Figure 3*. Next, we assessed whether systemic innate immunity was also suppressed. First, we noted that there were no obvious changes in the bone marrow (see Supplementary material online, *Figure S4C–G*). However, while circulating immune cell numbers were similar in all groups (see Supplementary material online, *Figure S4H*), abatacept prevented the increased representation of total and ‘inflammatory’ Ly6C^Hi^ and ‘reparative’ Ly6^Lo^ monocytes without influencing the neutrophil response post-CIRI (*Figure 5I–K* and Supplementary material online, *Figure S4I*). Moreover, while both monocyte subsets were decreased, the relative abundance of Ly6C^Lo^ vs. Ly6C^Hi^ cells was not altered (*Figure 5L*).

Combined, these data show that abatacept prevents T cells from contributing pro-inflammatory cytokines to the cardiac and mLN microenvironments post-CIRI, dampening local and systemic innate immune cell responses and thus minimizing the well-documented ability of several protagonists of cardiac damage and loss of cardiac function.

**Figure 6:**
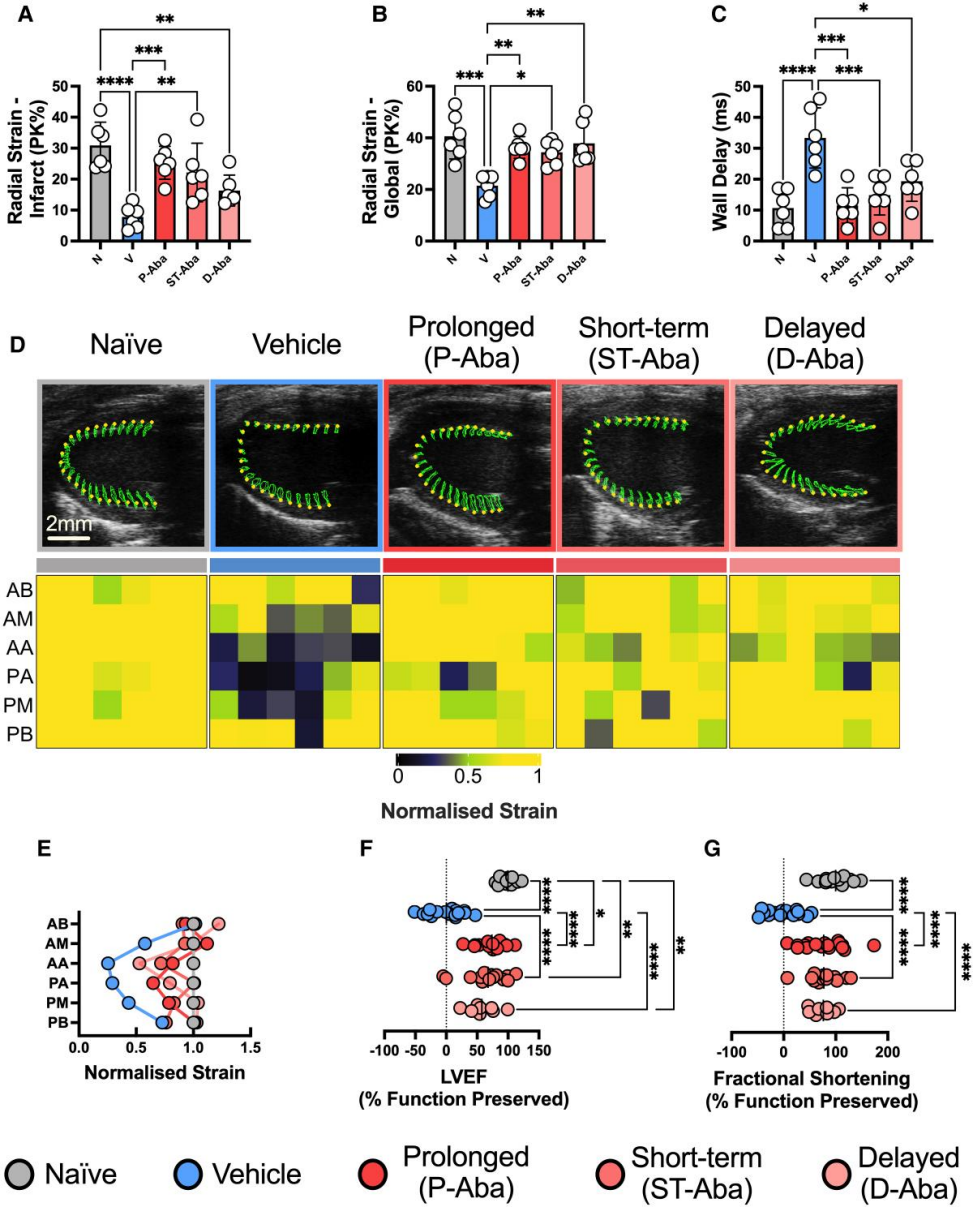
Global cardio-protection is conferred with prolonged, short-term, and delayed abatacept treatment post-MI. The cardio-protection provided by vehicle, prolonged, short-term and delayed treatment approaches was compared. Naïve mice not subjected to LAD ligation were included as controls. Radial strain at the anteroapical infarct site (*A*), globally (*B*), and maximum wall delay time (*C*). Representative echocardiographic images from radial strain analysis alongside heatmap of region-specific radial strain normalized to naïve function (*D*). Scale bar = 2 mm and applies to all representative images. Line graph of average normalized strain per segment from *D* (*E*). AB, anterior base; AM, anterior mid; AA, anterior apex; PA, posterior apex; PM, posterior mid; PB, posterior base. Percentage of LVEF (*F*) and fractional shortening (*G*) preserved by each treatment strategy. Vehicle control data are from the prolonged treatment cohorts, as these received the greatest number of vehicle injections. *n* represents the number of biological replicates, which are graphed as individual points where possible. *n =* 6 (*A–E*); *n* = 9–17 (*F–G*). Statistics used: ordinary one-way ANOVA tests were used. *P* < 0.05*; *P* < 0.01**; *P* < 0.001***; *P* < 0.0001****, ns = not significant. For visual clarity, only significant differences are shown; all other comparisons were not significantly different. N = Naïve, V = Vehicle, P-Aba = Prolonged Abatacept, ST-Aba = Short-term Abatacept, D-Aba = Delayed Abatacept.

As all treatment strategies were successful, we next directly compared the outcomes achieved with each approach. Starting with the most sensitive measure, we identified that delayed treatment did not significantly improve strain at the anterior apex (AA) in contrast to the prolonged and short-term treatments, despite qualitative improvement (*Figure 6A*). While this suggests, as might be expected, that more aggressive and timely treatment provides the greatest benefit, we found all treatment approaches provided significant improvements to global strain and maximum opposite wall delay time (*Figure 6B* and *C*). Closer inspection of region-specific strain further demonstrated the broad loss of function across the heart in vehicle-treated mice, which was prevented by abatacept treatment (*Figure 6D* and *E* and Supplementary material online, *Videos S1*–S5). Moreover, by quantifying the proportion of function retained using each treatment strategy, we further demonstrated the consistent preservation of cardiac function by abatacept with all three therapeutic strategies (*Figure 6F* and *G*). Collectively, these data support that even short-term and delayed abatacept treatment remains highly effective and may prove to be a robust approach for preventing MI- and CIRI-induced loss of cardiac function.

## 4. Discussion

Our understanding of the immunological mechanisms underlying cardiac tissue injury is only just emerging. However, it is clear that a better mechanistic comprehension is necessary in order to develop novel therapeutic approaches.^45,46^ The importance of this is emphasized by the fact that there are currently no therapeutics available to reduce the MI-induced inflammation and CIRI that exacerbate myocardial damage and drive poorer long-term outcomes. In this study, we have utilized the immuno-modulatory drug abatacept (CTLA-4-Ig) to explore the contribution of T cells to CIRI and their amenability to therapeutic modulation. The impact of this work is two-fold. Firstly, to our knowledge this is the first report highlighting a critical mechanistic role for T cell co-stimulation following MI with reperfusion. Moreover, from a therapeutic perspective, this research identifies abatacept as a promising candidate for preserving cardiac function in MI patients as an adjunct to recanalization.

In line with existing data,^38^ we have demonstrated significant activation of T cells in reperfused MI. This response was strongly biased toward the CD4^+^ compartment and dominated by a pro-inflammatory TNFα and IFNγ-producing T_H_1 phenotype. As T cell activation is detectible by day 3 post-MI, as seen here and by others,^28^ it is possible that innate-like or memory T cell responses are involved in addition to *de novo* activation of naïve cells. As such, the extent to which each response type contributes to cardiac damage and/or loss of function may be most important. Following abatacept treatment, T cell activation in the heart and draining mLN was almost completely prevented, demonstrating clear reliance on CD28:CD80/86 mediated co-stimulation. Co-stimulation is a critical checkpoint for the activation of naïve T cells on which memory T cell responses can be less reliant.^47–49^ This indicates that *de novo* activation is likely dominant post-MI and therefore potentially the best therapeutic target. Importantly, these data demonstrate a fundamental mechanistic role for this prototypical co-stimulation pathway in T cell activation following MI with reperfusion.

While *de novo* responses may dominate, a key outstanding question remains as to what MI-induced antigens T cells respond to. Current data demonstrate that heart-specific autoreactive T cells can escape from the thymus^50,51^ with the ability to differentiate into pro-inflammatory phenotypes and breach immunological tolerance.^52,53^ The most widely studied antigens are derived from myosin heavy chain, which appear able to drive both protective and pathogenic T cell responses in a context- and/or peptide-specific manner.^52–55^ The reliance of the T cell response on co-stimulation that we have identified, alongside significant up-regulation of CD69, which is strongly induced by T cell receptor–mediated antigen recognition, further supports a key role for antigen-specific T cell activation. Numerous studies have also demonstrated that enhancing T cell responses with ICI can lead to cardiotoxicity in humans and mice, reflecting a clear propensity for T cells to elicit cardiac-specific self-reactive responses.^13–16^ Indeed, there is growing evidence that such autoimmune (-like) responses could be a central feature of cardiovascular diseases in general,^10,11^ and we anticipate that these may be particularly important post-MI. If this hypothesis is correct, inhibiting T cell–mediated immunity may be critical to preventing long-term cardiac inflammation and increased cardiovascular risk.

A central aim of this study was to assess the therapeutic benefit of T cell inhibition. To do so, we utilized extensive echocardiography-based phenotyping in mice, including strain analysis. While not often applied in pre-clinical studies, strain analysis is relied on clinically to assess for abnormalities in contractility across the cardiac cycle. This approach has several major advantages, including increased sensitivity for cardiac dysfunction and a higher predictive value when it comes to cardiovascular events and the development of heart failure.^33^ Focusing on strain analysis of the primary infarct site (the AA), we observed the greatest preservation of function with prolonged abatacept treatment, with short-term treatment demonstrating slightly reduced but still significant therapeutic efficacy. While the delayed strategy did not provide a statistically significant improvement at the infarct site, there remained a ∼50% improvement in function in comparison to vehicle-treated mice (7.83 vs. 16.31 mean PK% respectively; relating to *Figure 6A*). Despite variation in this highly sensitive infarct-site specific analysis, global heart function was equally preserved by all treatment approaches. Critically, this demonstrates that most of the benefits from abatacept can be conferred even when treatment is delayed well beyond the typical timeline for PCI.^37^ Moreover, this also indicates potential to refine a short-term and/or delayed treatment strategy to achieve maximal protection even at the infarct site.

Interestingly, we identified that the protection we observed appears to be independent of total fibrotic burden. This further indicates that inflammation on its own is likely sufficient to cause significant reductions in cardiac function, as is supported by several other reports.^20,34–36^ This raises important questions as to the relative contributions of fibrosis-dependent and independent mechanisms of cardiac damage and reduced function. Intertwined with this are also important differences between reperfused and non-reperfused pre-clinical models of MI and the patient populations they ultimately reflect. Scarring in models of reperfused MI is far less severe than in the more commonly used permanent LAD ligation models, reflecting the therapeutic value of timely PCI.^56^ As such, the contributions of fibrotic vs. non-fibrotic mechanisms of reduced cardiac function and their magnitude may be very different depending on the model used. For example, the transfer of regulatory T cells post-MI conferred a ∼20% improvement to ejection fraction following CIRI, but a ∼50% improvement following permanent ligation.^57^

In our experiments, we anticipate that replacement fibrosis driven by the early events of cardiomyocyte death, where ‘time is myocardium’, dominates. However, direct inhibition of CD28 using an experimental blocking antibody has also been shown to be protective following permanent LAD occlusion without reperfusion.^58^ As in our study, this protection was associated with reduced cardiac inflammation. Overall, this suggests that T cell inhibition could still be leveraged in patients where reperfusion is not possible and fibrosis may play a more dominant role in the reduced function. Moreover, abatacept has been shown to be highly beneficial for the pre-clinical treatment of cardiac disease of multiple aetiologies, now inclusive of heart failure, myocardial necrosis, myocarditis and CIRI.^20–26^ This then suggests a common negative impact of T cell–driven inflammation across a spectrum of cardiac disease and potentially provides a unified target for adjunct cardiovascular therapies. Importantly, the dose of abatacept used by us and others in these pre-clinical settings is consistent with that used clinically (∼10 mg/kg) which may assist to streamline translational efforts.^19–22^

While T cells can clearly drive cardiac inflammation, their net contribution to loss of cardiac function post-MI has been unclear. In our study, vehicle-treated mice lost ∼37% of their LVEF, while abatacept-treated mice had a far smaller deficit (∼12–16% depending on treatment strategy). This suggests that over half of the functional loss in CIRI is dependent on the T cell response. Parallel to the direct impacts T cells can mediate through their expression of inflammatory cytokines (e.g. TNFα and IFNγ), T cells may promote further loss of function through the orchestrated recruitment of other immune cells. An excellent indicator of this is our important finding that T cell inhibition led to concomitant decreases in total and inflammatory cardiac monocytes, as well as those circulating systemically. Moreover, we still observed protection when abatacept intervention was delayed, precluding impacts on the acute inflammatory response within the first 24 h. As such, our data then also show that a sustained innate response post-MI is T cell dependent and positions T cells as central players in the CIRI response.

In respect to clinical data, there is a paucity of clinical trials on this topic beyond the assessment of cyclosporin, a widely used immunosuppressive drug which can reduce T cell responses, with the two studies having conflicting results.^59,60^ Changes in trial design, cyclosporin origin, and off-target effects have all been postulated as reasons for these contradictory findings. Additionally, different dosing regimens of cyclosporin appear to induce contradictory effects,^61^ with the relatively low dose (2.5 mg/kg) used in the aforementioned trials being potentially insufficient for robust and reproducible therapeutic T cell inhibition in the context of MI. To address this, a phase 3 study was registered; however, at the time of writing, results have not yet been posted (NCT02934217).

Despite the sparsity of clinical trials, there are promising insights from retrospective meta-analyses and population studies. Ozen *et al.*,^62^ for example, demonstrated a significant reduction in the risk of cardiovascular events in rheumatoid arthritis patients treated with abatacept, with this effect being predominantly attributed to a reduction in the incidence of MI.^62^ Several other studies have also supported this conclusion.^63–65^ It is important to consider that ‘anti-inflammatory’ studies in the cardiovascular setting most often focus on primary and secondary prevention of cardiac events, rather than attenuating MI/CIRI-induced inflammation or preserving cardiac function. As such, data on heart function in such trials is often missing. Moving forward, to achieve the maximal benefit for patients, both the prevention of MI and post-MI cardio-protection should be aimed for and closely monitored.

It is pertinent to also acknowledge that there has been significant concern in the field due to the lack of clinical translation from pre-clinical models of MI. This is a multifactorial problem that has been expertly reviewed elsewhere.^5,66,67^ Cellular responses such as cardiomyocyte death and early recruitment of innate immune cells begin early following coronary occlusion and hence strategies targeting these pathways are likely to have a very short time-to-treatment window, typically within a few hours of reperfusion and <12 h post-MI.^68^ We agree that therapies targeting these very acute consequences of MI are likely more challenging to translate, primarily because the molecular events mediating damage may already have occurred prior to treatment. Our approach differs by targeting the far slower dynamics of the adaptive immune response, which alleviates some of these time restrictions. Our data ultimately show that abatacept treatment within 24 h, in-keeping with typical in-patient stays for individuals with successfully reperfused MI (∼3 days), provides significant cardio-protection even where the treatment is delayed long after reperfusion.

Recognition of the potential advantages of targeting adaptive immunity following an MI can be seen in recent clinical trials. Zhao *et al*.,^69^ for example, conducted a phase 1/2a trial (RITA-MI; NCT03072199) to assess the safety of rituximab for the treatment of STEMI. Following the success of this trial, which confirmed safety and B cell suppression, RITA-MI 2 (NCT05211401) is now recruiting as a multi-centre phase 2b trial to assess directly whether rituximab can improve left ventricular function and cardiac remodelling post-STEMI.

In addition to preventing detrimental adaptive immune activation, there is also increasing interest in the promotion of protective regulatory T and B cell responses. Recently, results from the Phase I/II LILACS trial (NCT03113773) reported that treatment of stable coronary artery disease patients with IL-2 safely expanded T_regs_, with future studies now required to assess the clinical efficacy of this approach.^69^ Considering we find that abatacept also inhibits T_regs_ post-CIRI, these efforts raise a clear opportunity to improve T cell immunomodulation by also providing adjunct therapeutics like IL-2 to preserve the T_reg_ response and maximize anti-inflammatory protection.

In summary, this work identifies a new strategy for the prevention of CIRI via the therapeutic inhibition of T cell responses. We demonstrate a key role for CD28:CD80/86 mediated co-stimulation in the activation of T cells post-MI with reperfusion and a significant contribution from T cells to loss of cardiac function. We highlight that the protective effects of abatacept occur within one week, resulting in near-normal cardiac function. Importantly, our data support that abatacept is beneficial when administered at clinically feasible time points, either during PCI or within 24 h of MI. We believe this logistical compatibility makes targeting the adaptive immune response post-MI a strategy with higher likelihood of successful clinical translation. The role of the immune system in cardiovascular disease is unquestionable, yet recent revolutionary advances in immuno-modulatory therapies have had essentially no integration into cardiovascular medicine. The growing understanding of links between immunity and cardiovascular disease presents a tantalizing opportunity to re-create the transformational improvements in outcomes seen for cancer and autoimmune diseases in the cardiovascular sphere. Ultimately, abatacept represents a widely available and well-tolerated therapeutic that could be rapidly repurposed for the treatment of MI toward reducing the significant social, economic, and health burdens attributed to loss of cardiac function and the resulting heart failure.

Translational perspective There is a significant need for cardioprotective therapies that inhibit the overshooting immune-inflammatory responses causing cardiac ischemia-reperfusion injury following myocardial infarction (MI). To increase the chance of successful translation, the repurposing of approved, well characterized, and highly specific drugs is an attractive opportunity due to more favourable pathways to clinical approval. We demonstrate pre-clinically that treatment with abatacept, a highly specific FDA-approved biologic T cell inhibitor, prevents cardiac T cell activation and significantly preserves heart function post-MI. These findings support abatacept and T cell inhibition as a rapidly translatable therapy towards improved treatment of patients with MI.

## Supplementary Material

cvaf165_Supplementary_Data

## Data Availability

The data underlying this article will be shared on reasonable request to the corresponding author.
