# Supplementary material for: CTLA-4-Ig therapy preserves cardiac function following myocardial infarction with reperfusion
Source: Cardiovasc Res. 2025 Oct 3;121(13):2082–94. doi: 10.1093/cvr/cvaf165 (PMC12560771; doi:10.1093/cvr/cvaf165)
Supplement: cvaf165_Supplementary_Data [file cvaf165_supplementary_data.zip › 3) Noonan_&_Prijaya_Sup_Materials_CVR_R1.pdf]

CTLA-4-Ig Therapy Preserves Cardiac Function Following Myocardial Infarction with Reperfusion

Jonathan Noonan*#[1,3,4], Shania A. Prijaya*[1-3], Laura A. Bienvenu[2], Nalin H. Dayawansa[1,5,6], Marcel Michla[1], Viktoria Bongcaron[1,2], Prerna Sharma[1], Yuyang Song[2,3], Angela Huang[1], Anastasia Barbaro-Wahl[1], Yilu Huang[1], Hung Nguyen[1,2], Anne Nguyen[2], Andrew J. Murphy[3,7], Yiyu Zhang[3,7], Man Kit Sam Lee[7], Chad Johnson[8], Anna M.D, Watson[1,9], Anita C. Thomas[10,11], James D. McFadyen[1,3,12-14], Daniel G. Donner[3,5,10,11], Xiaowei Wang^[2,3,5,14], Karlheinz Peter^#[1,3-6,14]

*equally contributing First Authors

#Corresponding Authors

^equally contributing Senior Authors

AFFILIATIONS

[1]Atherothrombosis and Vascular Biology Laboratory, Baker Heart and Diabetes Institute, Melbourne, Australia

[2]Molecular Imaging and Theranostics Laboratory, Baker Heart and Diabetes Institute, Melbourne, Australia

[3]Department of Cardiometabolic Health, University of Melbourne, Melbourne, Australia

[4]Department of Immunology, School of Translational Medicine, Monash University, Melbourne, Australia

[5]Department of Medicine, School of Translational Medicine, Monash University, Melbourne, VIC, Australia

[6]Department of Cardiology, The Alfred Hospital, Melbourne, Australia.

[7]Haematopoiesis and Leukocyte Biology Laboratory, Baker Heart and Diabetes Institute, Melbourne, Australia

[8]Bioimaging Platform, La Trobe University, Melbourne, Australia

[9]Department of Diabetes, School of Translational Medicine, Monash University, Melbourne, Australia

[10]Translational Cardiology Centre, Baker Heart and Diabetes Institute, Melbourne, Australia

[11]Heart Centre, The Alfred Hospital, Melbourne, Australia

[12]Department of Clinical Haematology, School of Translational Medicine, Monash University, Melbourne, Australia

[13]Australian Centre for Blood Diseases, Monash University, Melbourne, Australia

[14]Baker Department of Cardiovascular Research, Translational and Implementation, La Trobe University, Melbourne, Australia

SUPPLEMENTARY FIGURES

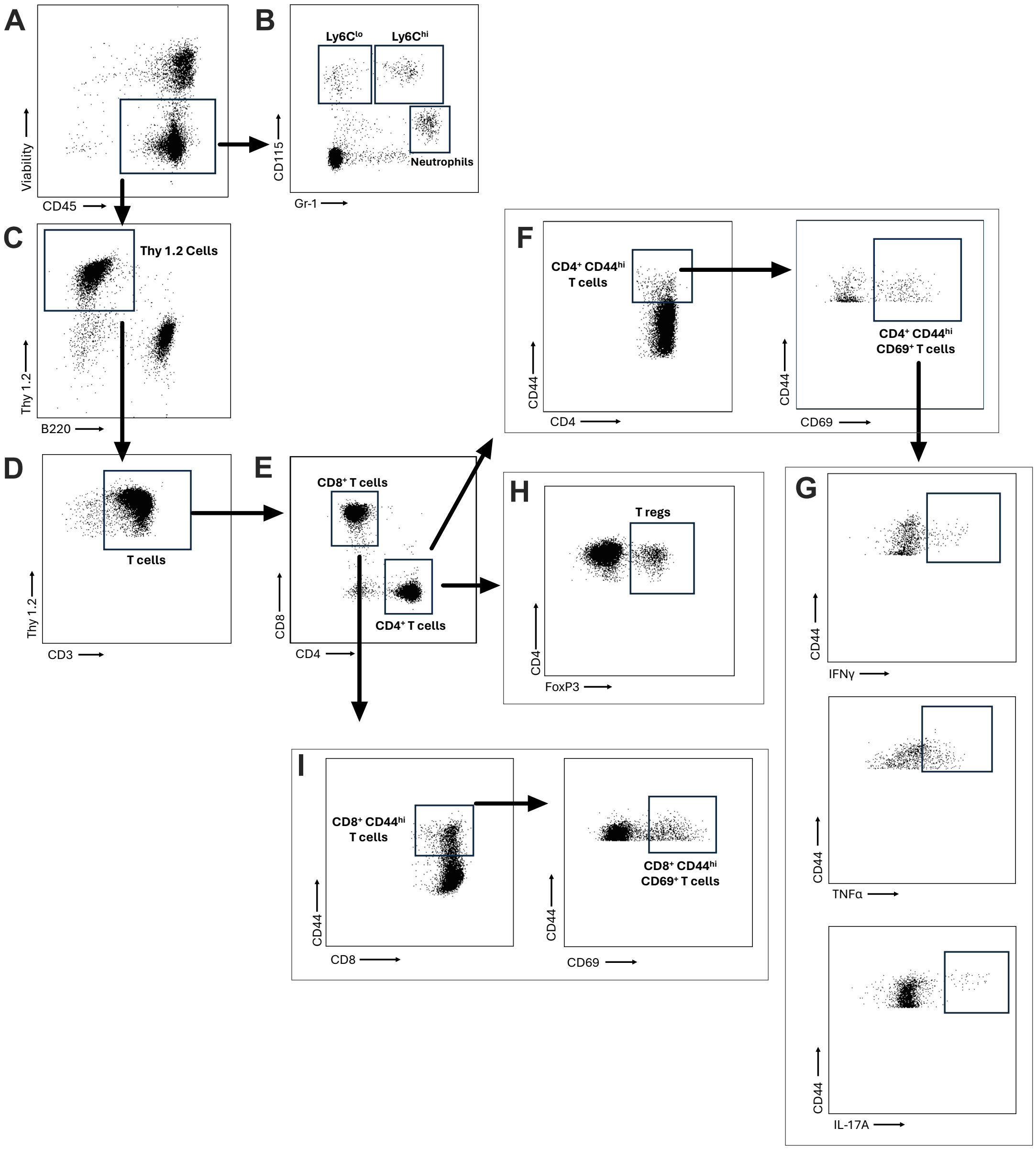

Supplementary Figure 1: Overview of flow cytometry gating strategies. Live CD45^{+} cells were initially gated (**A**). Neutrophils (CD115^{-} Gr-1^{+}) and monocytes (CD115^{+}) were identified, with monocytes further stratified based on Ly6C^{lo} or Ly6C^{hi} expressing using a dedicated Ly6C antibody or using Gr-1 (**B**). T cells were then identified based on CD3 expression with pre-gating for Th1.2 in intracellular staining panels (**C & D**). CD4^{+} and CD8^{+} T cells were then investigated (**E**). Effector/memory CD4^{+} CD44^{hi} T cells and their expression of CD69^{+} was quantified (**F**). These cells were then interrogated for their ability to produce IFNγ, TNFα and IL-17A (**G**). Regulatory CD4^{+} T cells were identified based on FoxP3 expression (**H**). Effector/memory CD8^{+} CD44^{hi} T cells and their expression of CD69^{+} was quantified (**I**).

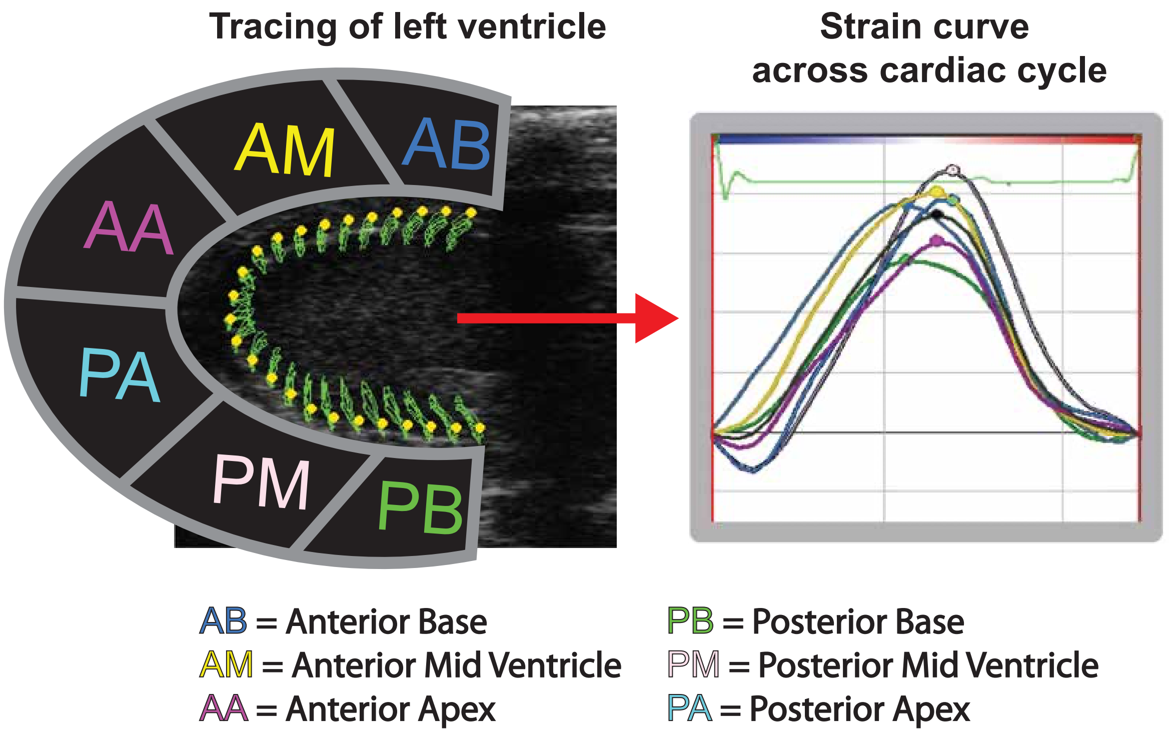

Supplementary Figure 2: Overview of strain analysis. Following echocardiographic imaging, the muscle of the left ventricle is traced within the Vevo software. Using the strain analysis feature, the heart is then automatically segregated into the key anatomical regions of the heart (AB, AM, AA, PB, PM, PA), with multiple speckle tracking points present within each region. These tracking points are then analysed for the synchronicity of their movement across the cardiac cycle, creating a curve which conveys whether the cardiac regions are moving synchronously (e.g. Supplementary Video 1), asynchronously or with reduced movement (e.g., Supplementary Video 2).

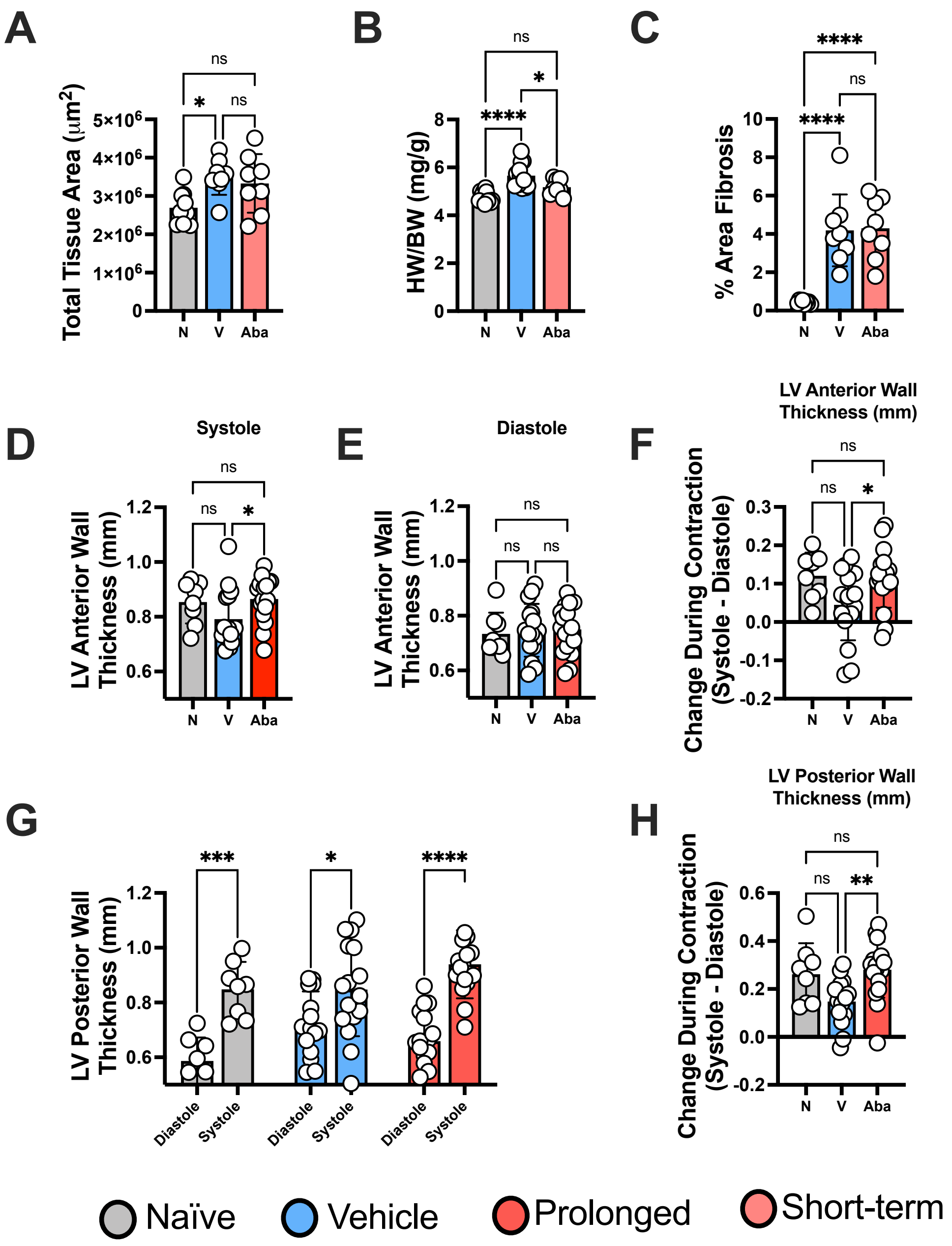

Supplementary Figure 3: Cardiac remodelling and contraction following abatacept treatment. C57BL/6 mice were subjected to temporary LAD ligation and then treated with abatacept or vehicle using the short-term (**A–C**) or prolonged treatment regime (**D–F**). Tissue area of mid ventricular sections assessed by histology (**A**). Heart weight (HW) to body weight (BW) ratios (**B**). Proportional representation of fibrosis assessed by picrosirius red staining (**C**). Left ventricle (LV) anterior wall thickness at systole (**D**) and diastole (**E**). Change of LV anterior wall thickness between systole and diastole (**F**). Paired analysis of LV posterior wall thickness for naïve, vehicle and abatacept treated mice (**G**). Change of LV posterior wall thickness between systole and diastole (**H**). *n* represents the number of biological replicates, which are graphed as individual points. *n = 8-17*. Statistics used: Ordinary one-way (**A,B,C,E,F,H**) and Brown-Forsythe and Welch (**D**) ANOVA and Mixed effects analyses (**G**) were performed. $p<0.05$*; $p<0.01$**; $p<0.001$***, $p<0.0001$****, ns = not significant.

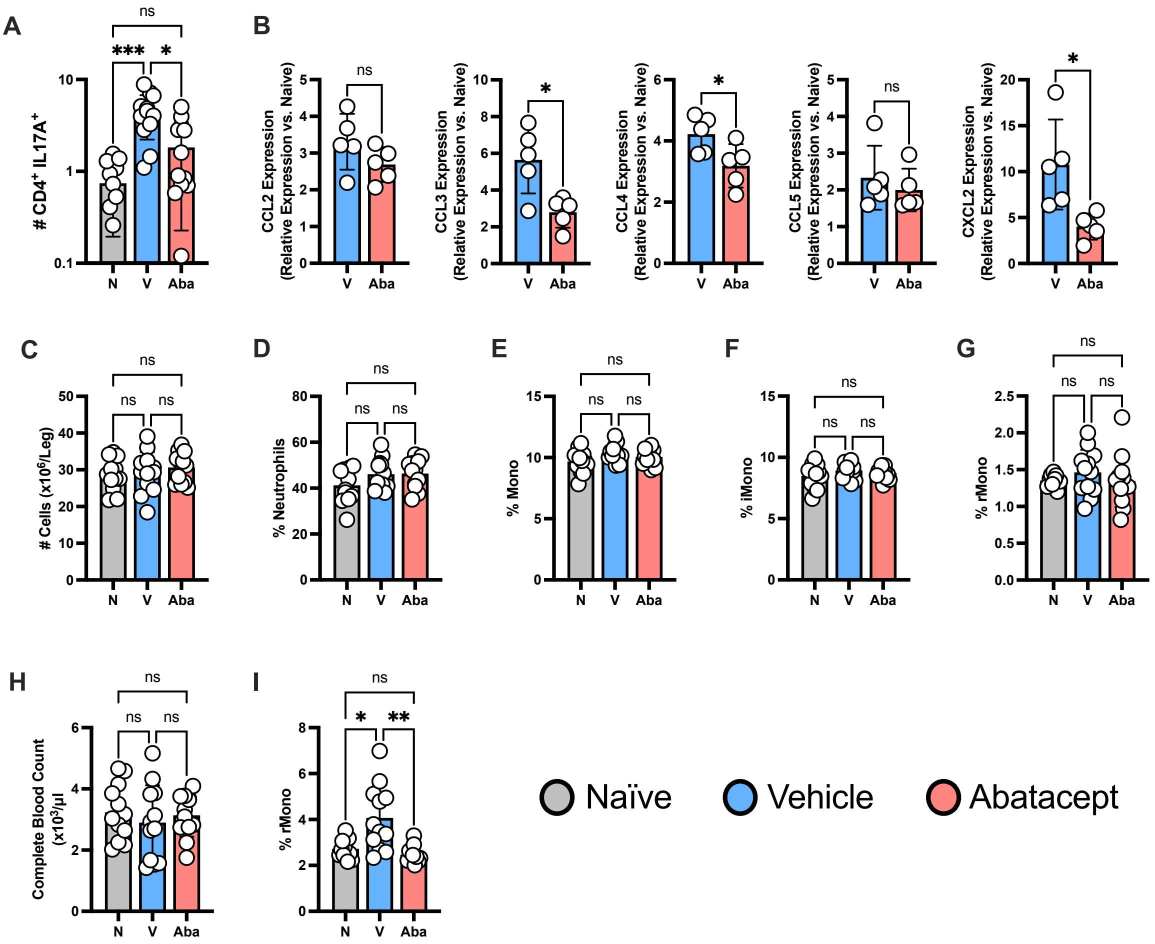

Supplementary Figure 4: Local and systemic immune suppression following abatacept treatment. C57BL/6 mice were subjected to temporary LAD ligation and then treated with abatacept or vehicle using a short-term treatment regime. Naïve mice not subjected to LAD ligation were included as controls. All analyses are from 7 days post-CIRI. Numbers of activated IL17 producing $CD4^{+}CD44^{+}CD69^{+}$ T cells in the mLN (**A**). Normalised expression of *CCL2*, *CCL3*, *CCL4*, *CCL5* and *CXCL2* in magnetically sorted cardiac $CD45^{+}$ cells, expressed relative to expression in naïve (**B**). Total number of cells isolated from the bone marrow (**C**). Proportion of neutrophils (**D**), total monocytes (**E**), 'inflammatory' $Ly6C^{Hi}$ monocytes (**F**), 'reparative' $Ly6C^{Lo}$ monocytes [rMono] (**G**) in bone marrow. Total number of circulating immune cells (**H**) and proportion of $Ly6C^{Lo}$ monocytes (**I**). *n* represents the number of biological replicates, which are graphed as individual points where possible. *n* = 5-12. Statistics used: Brown-Forsythe and Welch (**A-I**) and Ordinary one-way (**C-H**) ANOVA and unpaired t-tests (**B**) were performed. $p<0.05$*; $p<0.01$**; $p<0.001$***, ns = not significant.

Supplementary Videos 1-5: Representative *in vivo* echocardiography assessment of Naïve (1) and Vehicle-treated mice (2), as well as Abatacept treated mice from the Prolonged (3), Short-term (4) and Delayed (5) regimens. All images are from week-4 post CIRI, parasternal-long-axis view via B-mode.

Supplementary Table 1: Antibodies and Dyes.
The complete list of antibodies/dyes used in the study and their working concentrations.

Target antigen / dye	Vendor/source	Category #	Working concentration
Fixable Viability Dye eFluor^{TM} 780	ThermoFisher Scientific	65-0865-14	1/1000
Fixable Viability Dye eFluor^{TM} 506	ThermoFisher Scientific	65-0866-18	1/1000
Fixable Viability Stain 700	BD Biosciences	564997	1/2000
CD45	BD Biosciences	564279	1/200
CD45	BioLegend	103126	1/400
CD3e	Abcam	Abcam16669	1/200
CD3 Complex	BD Biosciences	560527	1/100
CD90.2 (Thy1.2)	BioLegend	140324	1/400
CD4	BD Biosciences	560782	1/100
CD8	BD Biosciences	563332	1/200
CD44	BD Biosciences	563970	1/200
CD69	BD Biosciences	560689	1/100
FoxP3	Invitrogen	48-5773-82	1/100
IFNγ	BioLegend	505813	1/400
TNFα	BioLegend	506328	1/400
IL17A	BioLegend	506912	1/400
Ly6C	BD Biosciences	553104	1/100
CD68	Bio-Rad	MCA1957	1/200
CD115	Invitrogen	12-1152-82	1/400
Gr-1	BD Biosciences	552093	1/400
Streptavidin-HRP	Abcam	Abcam7403	1/200

Supplementary Table 2: Reagent Kits.

Reagent/Kit	Vendor	Category #
Cell Activaton Cocktail (with Brefeldin A)	BioLegend	423303
BD Cytofix/Cytoperm™ Fixation/Permeabilization Kit	BD Biosciences	554714
EasySep™ Mouse CD45 Positive Selection Kit	Stem Cell Technologies	18945
TRIzol	ThermoFisher Scientific	15596026
iTaq™ Universal SYBR® Green One-Step Kit	Bio-Rad	172515

Supplementary Table 3: Primer Sequences used for qPCR.

Gene name	Sequence
CCL2	Forward: TTAAAAACCTGGATCGGAACCAA
	Reverse: GCATTAGCTTCAGATTTACGGGT
CCL3	Forward: TTCTCTGTACCATGACACTCTGC
	Reverse: CGTGGAATCTTCCGGCTGTAG
CCL4	Forward: TTCCTGCTGTTTCTCTTACACCT
	Reverse: CTGTCTGCCTCTTTTGGTCAG
CCL5	Forward: GCTGCTTTGCCTACCTCTCC
	Reverse: TCGAGTGACAAACACGACTGC
CXCL2	Forward: CCAACCACCAGGCTACAGG
	Reverse: GCGTCACACTCAAGCTCTG
GAPDH	Forward: AGGTCGGTGTGAACGGATTTG
	Reverse: TGTAGACCATGTAGTTGAGGTCA

SUPPLEMENTARY METHODS

Mice

Male and female 6-14 week old C57BL/6J mice (Jackson Laboratories, USA) were bred at the Alfred Medical Research and Education Precinct (AMREP) Animal Services facility and randomly assigned to different treatment strategies. All experiments involving animals were approved by the Alfred Alliance Animal Ethics Committee (E/1970/2020/B & P8270) and conformed to the guidelines under Directive 2010/63/EU of the European Parliament on the protection of animals used for scientific purposes.

Cardiac Ischemia-Reperfusion Injury Model

MI was induced as previously described.[1] Animals were anesthetized with ketamine (10 mg/kg), xylazine (5 mg/kg), and atropine (1 mg/kg) intraperitoneally (i.p.). Mice were endotracheally intubated with a respirator set to 0.18 ml at 120 breaths per minute. Carprofen (5 mg/kg) was injected subcutaneously (s.c.) following successful intubation to provide post-surgical analgesia. The surgical site was then sterilised with povidone-iodine and an incision was made on the left side of the chest to expose the muscle layer. Bupivacaine (1 mg/kg, s.c) was injected to provide local anaesthesia at the incision area. An incision was then made at the intercostal space between the second and third ribs. The ribs were opened with a micro-extractor, exposing the thoracotomy region. A silk (7-0) suture was used to ligate and occlude the left anterior descending (LAD) artery. To allow for reperfusion, the ligation suture was tied through two exteriorized releasing loops. The chest cavity and skin were then closed, and mice injected with the anti-sedative atipamezole (200 ug/kg, s.c) and placed in recovery. Reperfusion was induced after 60 min by pulling the exteriorized loops, thus releasing the ligation, and allowing blood flow to continue. Mice were euthanised using ketamine (300

mg/kg) and xylazine (30 mg/kg). Humane killing was then confirmed using an appropriate secondary method.

Abatacept Treatment

Mice were randomised into different treatment groups and were given abatacept (Bristol Myer Squibb; 10 mg/kg) or vehicle control (0.9% sodium chloride) intraperitoneally (i.p). Prolonged treatment groups were given tri-weekly (Monday, Wednesday, Friday) injections from the time of reperfusion. Short-term treatment groups were given abatacept/vehicle injections at reperfusion and at 3 days post-MI. Delayed treatment groups were given abatacept/vehicle injections 1- and 4- days post-MI. Naïve mice were included as controls (as opposed to Sham surgery) given our focus on long-term functional / physiological variables and the cellular immune response which are not significantly influenced by the sham surgery in our experience and in published works.[2]

Echocardiography

Imaging and analysis were done by double-blinded investigators. Mice were placed under light sedation with 1–2% isoflurane on a heated VisualSonics imaging station. Following shaving, ultrasound was performed using a Vevo2100 high-resolution small-animal scanner (VisualSonics Inc., Canada) and 22–55 MHz MS 550D transducer (VisualSonics Inc., Canada). The parasternal short and long-axis views of the heart were imaged. All functional parameters were calculated from long-axis views, except for fractional shortening, which utilised short-axis B-mode images. Radial strain analyses were performed using a VevoStrain speckle-tracking algorithm (VisualSonics Inc., Canada). Baseline measurements were conducted 24-48 hours prior to CIRI surgery.

Flow Cytometry

Phosphate-buffered saline (PBS)–perfused heart tissue was finely minced before being transferred into an enzyme cocktail of Liberase TL (Sigma Aldrich, USA) and DNAse1 (Sigma Aldrich, USA) and placed into a 37°C water bath. Heart samples were triturated in the digestion mix every 15 min for a total of 45 min, ultimately yielding a single cell suspension. Heart-draining mediastinal lymph nodes (mLN) were mechanically homogenized through a 40 µm filter and resuspended in PBS. Blood was lysed with 1x RBC Lysis Buffer for 15min on ice. Bone marrow was flushed from one leg and lysed using 1x RBC Lysis Buffer for 5min on ice. For intracellular cytokine staining, cells were first stimulated with Cell Activation Cocktail with Brefeldin A (BioLegend, USA) for 3 hours at 37 ºC. Cells from all tissues were stained with fixable viability dye for 15 min at 4 °C. Extracellular antibody staining with fluorochrome-conjugated antibodies was then performed. Intracellular cytokine and transcription factor staining was then performed using the BD Cytofix/Cytoperm™ Fixation/Permeabilization Kit (BD Bioscience, USA) according to manufacturer instructions and stained with intracellular antibodies for 60 min at 4 ºC. Data were acquired from stained cells using a BD LSR Fortessa X-20 (BD Bioscience, USA). Cell numbers were presented relative to naïve mice for intracellular cytokine analyses. Data analysis was performed using FlowJo (Flowjo LLC, USA). A complete list of antibodies/dyes and reagents used is provided in Supplementary Tables 1 and 2, respectively.

Immunohistochemistry

Dewaxed 6 µm sections underwent heat antigen retrieval in citrate buffer, peroxidase block (0.3% H_2O_2/PBS), avidin-biotin block (Vector), non-specific binding block (10% normal horse serum (NHS; Vector)/PBS), and overnight incubation with CD3e antibody in 2% NHS/PBS. Sections were then incubated in biotinylated secondary antibody (Vector BA-4001 or BA-

1000). CD3e sections were incubated with streptavidin-HRP (Abcam, United Kingdom) before TSA, incubation with avidin-biotin complex (Vector PK-6100), visualisation with DAB (Sigma Aldrich, USA) and counterstaining with Mayer's hematoxylin.[3,4] Images were acquired with an Olympus BX43/DP28 camera (Olympus, Japan) or Zeiss Axioscan 7 (Zeiss, Germany). A complete list of antibodies used is included in Supplementary Table 1.

Analysis of Cardiac Fibrosis by Histology

Midsections of the left ventricle (LV) were fixed with 4% paraformaldehyde overnight and then embedded in paraffin blocks. The hearts were then serially sectioned with a Microm Rotary Microtome into 4 µm sections. Fibrotic cardiac tissue was then stained using Picrosirius Red (PSR). The percentage of cardiac fibrosis was calculated as the percentage of total PSR^+ area vs. total LV tissue area using the FIJI Image J Software (National Institute of Health, Bethesda, USA).

Gene Expression Analysis of Cardiac $CD45^+$ cells by qPCR

$CD45^+$ cells were purified from mouse hearts using the EasySep™ Mouse CD45 Positive Selection Kit (STEMCELL Technologies, Canada) according to the manufacturer's instructions. Total RNA was extracted from isolated cells using TRIzol (ThermoFisher Scientific, USA). Real-time quantification of gene expression (qPCR) was performed with the iTaq™ Universal SYBR® Green One-Step Kit (Bio-Rad, USA) using the QuantStudio 7 Flex system (ThermoFisher Scientifics, USA). The relative expression of each gene was normalized to GAPDH. The sequences of primers used were synthesized by Integrated DNA Technologies (USA). The complete list of reagents and sequences used are included in Supplementary Table 2 and 3 respectively.

Statistics

All statistical analyses were conducted using GraphPad Prism (Version 10.1.1). Outlier testing was conducted using the ROUT test followed by normality testing using Shapiro–Wilk. All unpaired data with two groups were normally distributed. For those with equal variance (defined by the F test), an unpaired Student's t test was used. In the absence of equal variance, a Welch's t test was used. For Figure 1F, comparing the number of CD4 versus CD8 T cells within mice, a paired Student's t-test was used. For data with more than two groups: normally distributed data with equal variance (defined by a Brown–Forsyth test) were assessed using a one-way ANOVA with Tukey's multiple comparison test; normally distributed data with non-equal variance were assessed using a Brown–Forsythe and Welch ANOVA with Dunnett's T3 multiple comparisons tests; non-normally distributed data were assessed using a Kruskal–Wallis with Dunn's multiple comparisons test. Grouped data were assessed using a mixed-effects model with Greenhouse–Geisser correction and Šidák's multiple comparisons test.

SUPPLEMENTARY REFERENCES

1. Ziegler M, Hohmann JD, Searle AK, Abraham M-K, Nandurkar HH, Wang X, Peter K. A single-chain antibody-CD39 fusion protein targeting activated platelets protects from cardiac ischaemia/reperfusion injury. *Eur Heart J* 2018;**39**:111–116.

2. Iyer RP, Castro Brás LE de, Cannon PL, Ma Y, DeLeon-Pennell KY, Jung M, Flynn ER, Henry JB, Bratton DR, White JA, Fulton LK, Grady AW, Lindsey ML. Defining the sham environment for post-myocardial infarction studies in mice. *Am J Physiol Heart Circ Physiol* 2016;**311**:H822-836.

3. Hübschle T, Thom E, Watson A, Roth J, Klaus S, Meyerhof W. Leptin-Induced Nuclear Translocation of STAT3 Immunoreactivity in Hypothalamic Nuclei Involved in Body Weight Regulation. *J Neurosci* 2001;**21**:2413–2424.

4. Krabichler Q, Vega-Zuniga T, Carrasco D, Fernandez M, Gutiérrez-Ibáñez C, Marín G, Luksch H. The centrifugal visual system of a palaeognathous bird, the Chilean Tinamou (*Nothoprocta perdicaria*). *J of Comparative Neurology* 2017;**525**:2514–2534.
